# Defining familiarity in nursing homes providing care for residents with dementia: a scoping review

**DOI:** 10.3389/frdem.2025.1470066

**Published:** 2025-02-03

**Authors:** Joanna Sun, Sumiyo Brennan, Therese Doan

**Affiliations:** ^1^Wicking Dementia Research and Education Centre, University of Tasmania, Hobart, TAS, Australia; ^2^Institute for Gerontology, J. F. Oberlin University, Tokyo, Japan; ^3^School of Nursing, San Francisco State University, San Francisco, CA, United States

**Keywords:** dementia, built environment (BE), familiarity, aged care, architecture, nursing, design

## Abstract

**Objective:**

This study explores the underpinning definitions associated with familiarity in the context of dementia inclusive design and nursing home environment.

**Background:**

Environmental design in nursing homes impacts the quality of life and care of residents with dementia. One of the key principles of design is the need for the environment to achieve a sense of familiarity. However, there are divergent perspectives regarding the term “familiarity.” Inconsistent definitions are challenging to understand, and they continue to impact the implementation of good design. To that end, this scoping review examines the definitions and associated with familiarity, dementia, and the design of nursing home environments.

**Methods:**

The Arksey and O'Malley methodological framework and the Preferred Reporting Items for Systematic Reviews and Meta-Analyses Extension for Scoping Reviews ensure the rigor of the scoping review. Searches were conducted using six databases for peer-reviewed publications in English from 1991 to 2023. Search terms included “dementia,” “nursing home,” “long-term care,” “familiar,” “familiarity,” and “environments.”

**Results:**

The searches yielded 245 publications. Of those, 46 studies from 19 countries were included in this review. The review uncovered three central themes and compelling evidence citing the inclusion of homelike environments. Emphasis on design considerations includes the wellbeing of the designated population and the need to embrace multisensory integration in the design process.

**Conclusions:**

A strong link exists between familiarity and key design considerations, such as a homelike environment, wellbeing, and multisensory integration. These considerations can enhance the effectiveness of the design of familiar environments.

## 1 Introduction

The design of the built environment significantly contributes to the quality of life (QOL) of people with dementia in nursing homes (Fleming et al., 2016; Nordin et al., 2017). A higher quality of life was closely tied to living in an environment that fosters a sense of familiarity and inclusion (Fleming et al., 2016). Including a familiar environment or familiarity is a key principle in optimizing the design of built environments for people with dementia in long-term care facilities (Fleming et al., 2023). In the context of dementia, familiarity can be defined as a psychological phenomenon that elicits a feeling of prior experience as an association with past encounters. Essentially, familiarity is a feeling that arises from the memory of past experiences that connects to previous encounters and helps trigger a sense of recognition (Bastin et al., 2019). It is theorized that familiarity underpins information recognition, including environments and objects. The integrative memory model by Bastin et al. (2019) provides valuable insights into the brain changes that affect the sense of familiarity in dementia, particularly in Alzheimer's Disease. As dementia progresses, cognitive decline often occurs, leading to difficulties remembering and recognizing specific items. However, even in the later stages of the disease, individuals can still connect non-related information with a sense of familiarity, showcasing a different aspect of memory retention (Bastin et al., 2019; Braak and Braak, 1991, 1995). This understanding highlights the complex nature of memory processes in dementia and emphasizes the need for tailored approaches to support those affected by this condition (Bastin et al., 2019). The need for familiarity is not an emerging concept. Literature highlighting the need for such an environment to support people with dementia can be found as far back as the early 1980s (Skolaski-Pellitteri, 1984; Small et al., 1981). However, despite the historical evidence and emphasis, the literature fails to adequately address the complexities and intricacies of awareness, planning, and implementation. A familiar environment encompasses multiple fragmented definitions, which can cause confusion in the design of environments. In some instances, the literature touches on a familiar environment being homelike, while others may only focus on the need to include familiar personal artifacts (Van Hoof et al., 2016). The design of nursing homes for residents with dementia also comprises a range of stakeholders who may all hold different views on understanding and defining a familiar environment. Stakeholders who are implementing the principle of familiarity stem from various sectors; they may be a combination of health workers, architects, administrators, each holding a different understanding of familiarity in design and dementia (Sun and Fleming, 2018). The culture of a population, their values, and beliefs can also shape the differences and disparities in how a familiar environment is implemented.

Implementation of dignified and enabling environments has emerged as a global priority recognized by Alzheimer's Disease International (ADI). In 2020, ADI released a report which focused on developing dementia-inclusive environments worldwide and a set of underpinning design principles (Fleming et al., 2020). Within the report, the term is used variably across contexts, reflecting multiple meanings. It has been described as part of a perception, aesthetic quality, principle, or a design element. This lack of precise definition causes ambiguity and implies various attributes or values that vary based on context. Establishing the definition of familiarity could enable a common understanding of how the term relates to dementia-inclusive design.

To create an inclusive and familiar nursing home environment for residents with dementia, stakeholders need a clear understanding of what familiarity entails. This scoping review seeks to consolidate the definitions, with the aim of providing a holistic understanding of familiarity and a unified perspective for all. This clarity will help with planning, implementation and evaluations, leading to better outcomes for people with dementia residing in nursing homes, care partners and stakeholders in the aged care sector.

## 2 Methods

A scoping review was found to be the most effective approach, as this is not a strategic assessment of the quality of literature on familiarity and nursing home environments for individuals with dementia, but a structured exploratory synthesis of the evidence (Booth et al., 2016, p. 329). This review can provide clarity as it examines the existing accepted definitions of the. The review was conducted following the steps outlined by Arksey and O'Malley (2005, Figure 1). The Arksey and O'Malley (2005) methodological framework was selected for this review as it can show a wide range of evidence, and it is ideal for quick evidence mapping rather than an in-depth analysis. Frameworks for in-depth analysis would be ideal for more well-researched topics.

**Figure 1:**
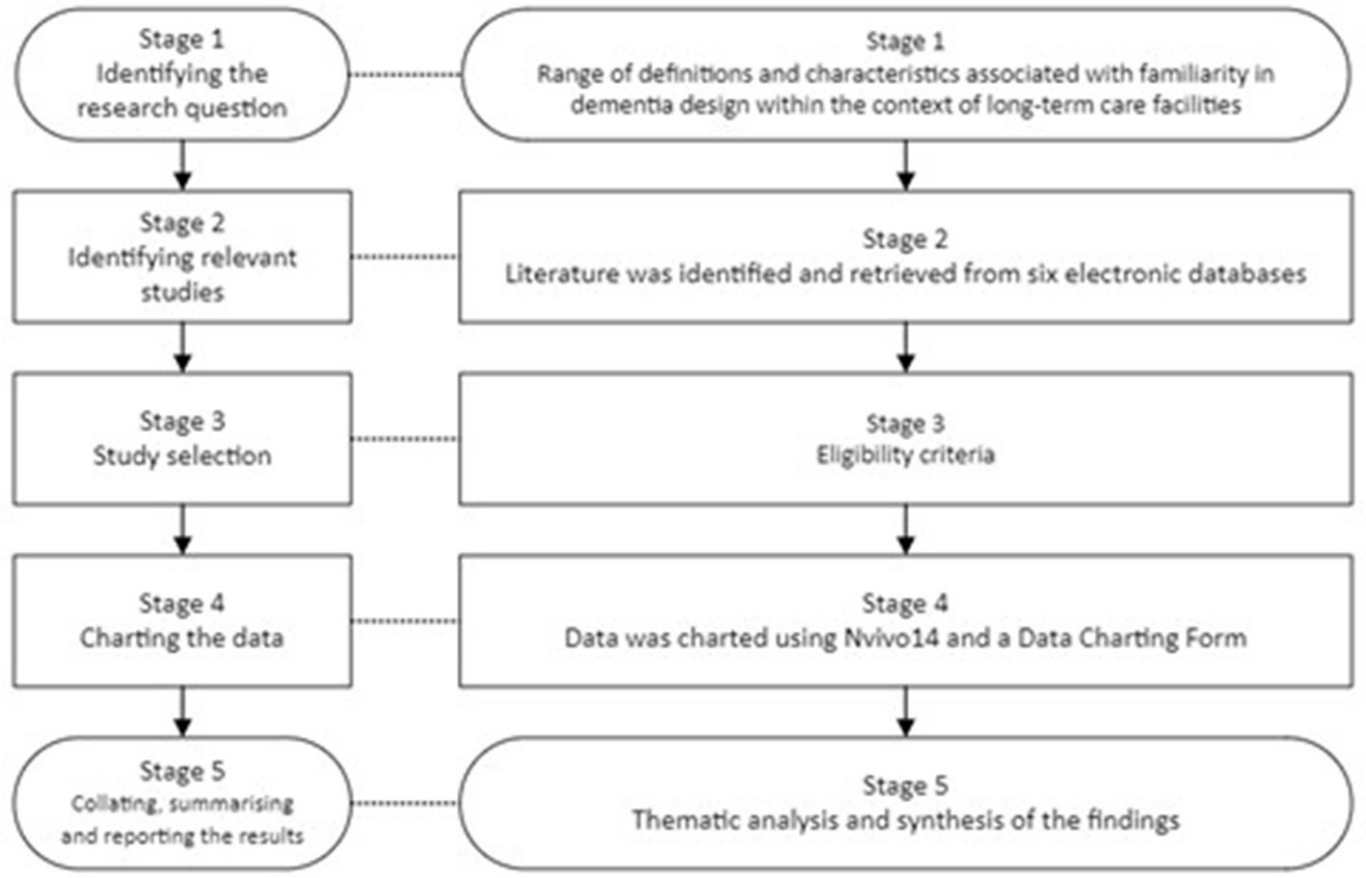
An outline of the scoping review within the Arksey and O'Malley methodological framework.

**Figure 2:**
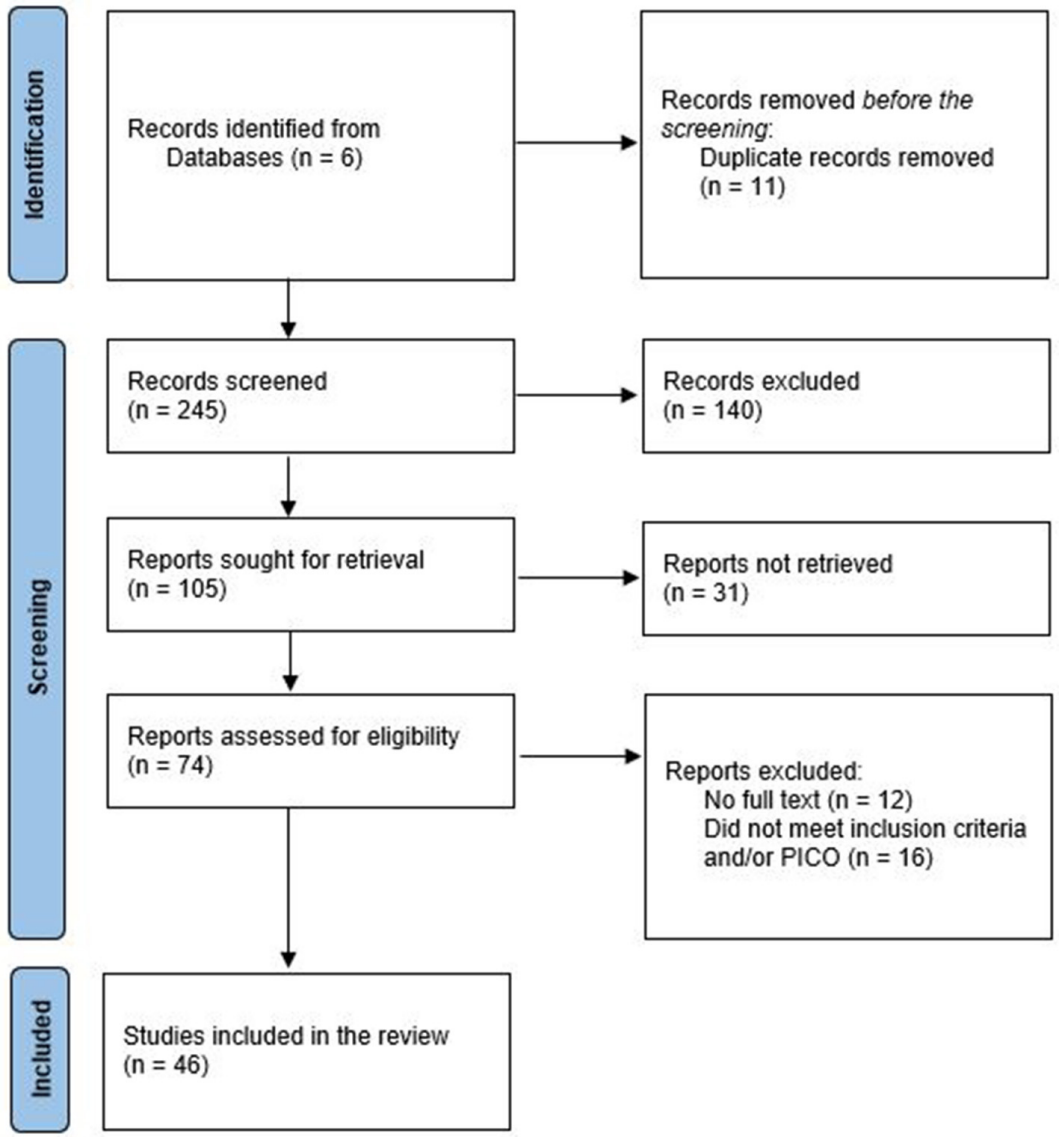
Preferred Reporting Items for Systematic Reviews and Meta-Analyses (PRISMA) flow diagram.

The research questions this scoping review seeks to address are, “how are familiarity or familiar environments within the context of nursing homes or LTC facilities defined in the existing literature, and what are the accompanying characteristics?” Literature was retrieved from six electronic databases: Cumulative Index to Nursing and Allied Health Literature (CINAHL), PubMed, PsycINFO, ScienceDirect, Scopus, and Web of Science. In addition to the Arksey and O'Malley framework (2005), the Preferred Reporting Items for Systematic Reviews and Meta-Analyses Extension for Scoping Reviews (PRISMA-ScR) provided a roadmap in the identification of the literature (Page et al., 2021). The PRISMA-ScR describes search results precisely and makes the results easier to understand visually by increasing relevance to decision-making (Tricco et al., 2018). An iterative team approach was employed to agree on the eligibility criteria. All authors in this study have carried out research design or have engaged in both research and implementation of dementia design. The following search terms were identified through the process, “dementia,” “nursing home,” “long-term care,” “familiar,” “familiarity,” and “environments.” Search terms were tested and modified, which resulted in the inclusion of “Special Care Units.” No gray literature was included in this review to ensure a strict alignment with the research question, which focuses on the existing scholarly literature. Only English language papers were included. This study utilized thematic analysis to integrate the findings across the studies. The process of the comparative thematic analysis consisted of three principle stages, (1) free line-by-line coding, (2) the organization of free code into themes, and the (3) generation of key themes (Booth et al., 2016). Before the analysis process, the authors familiarized themselves with the full-text literature (Braun and Clarke, 2021) before engaging in line-by-line coding using the NVivo 14 software (Lumivero, 2024). The codes were compared to capture significant themes across the data set and a codebook was created using NVivo14. The sub-themes and themes were then systematically defined and included in a Microsoft Excel (Microsoft Corporation, 2021) data charting form. The authors engaged in a review of the codes, sub-themes and themes that ensured alignment and coherence in the development themes with the research questions. The codes, sub-themes and themes are included in the charting form. The form also contained information such as the author, year of publication, country of study, design, and demographics of participants. The finalized version of the form was carried out by two researchers. A flow diagram (Figure 2) and a codebook containing all themes and codes were developed. The data charting form can be found at https://osf.io/q8yb7.

## 3 Results

**Figure 3:**
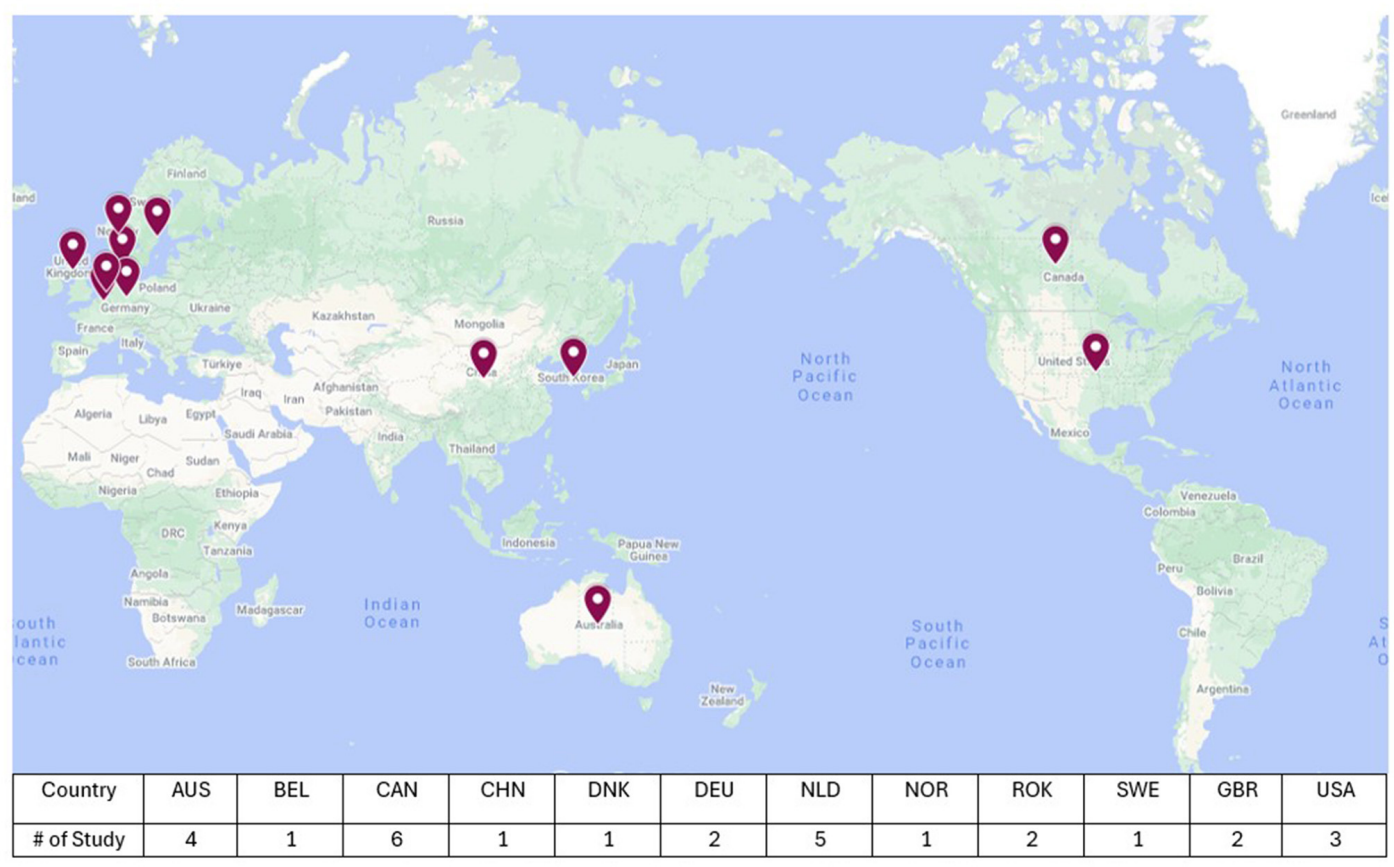
Country and number of publications from empirical studies (*n* = 27, Google My Map, 2024). Please note that some studies comprised more than one country (Lee et al., 2021; Roberts, 2023).

A total of 46 articles from 20 countries were reviewed, comprising of empirical studies (*n* = 27) and literature or scoping reviews (*n* = 19, Figure 3). The publications reviewed were from Canada (21%), Australia (14%), the United States of America (10%) and the United Kingdom (7%). Two publications included studies conducted in two countries (Lee et al., 2021; Roberts, 2023).

**Figure 4:**
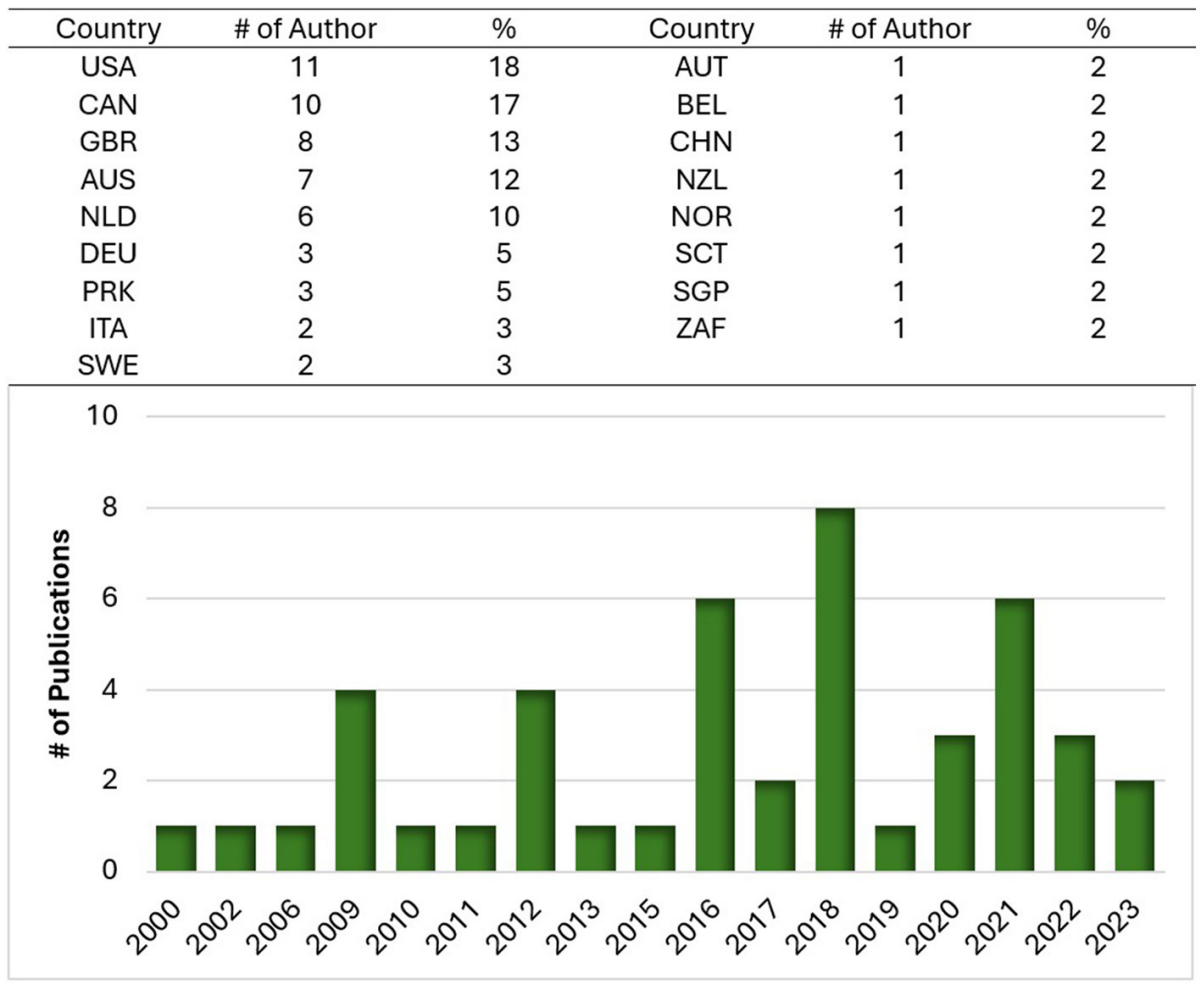
All authors' affiliation (*n* = 60) by country (*n* = 17) and year distribution of 46 published articles (2000–2023, *n* = 46).

Aside from the 19 papers that were literature or scoping reviews, it was found that many studies were published by researchers based in countries different from those where the studies were conducted. To provide an overarching view, Figure 4 shows the authors' affiliation countries and the distribution of publications over time, and there are clear trends between both Figures. Figure 3 presents the countries in which studies have been undertaken, and Figure 4 presents the countries in which the authors' affiliated institutes are located. It can be observed that key countries such as Canada, the United States of America, Australia, the United Kingdom, and Netherlands have engaged in research on environmental design in dementia with a lens of familiarity.

Clear definitions of familiarity were found in 63 % of the publications. In the context of the review, a clear definition consists of one that is easy to understand and actionable. It should contain a concise explanation of the terms, include fundamental characteristics or means of implementation, and exclude any ambiguous information. Three key themes; “a homelike environment, connection to wellbeing, and multisensory integration within the built environment defined a “familiar” environment. Together, these three themes describe a familiar care environment that feels like home, supports wellbeing, and integrates sensory elements to enhance comfort and reduce distress. This environment meets physical, emotional and psychological needs, creating a holistic, nurturing, and compassionate space that aligns perfectly with the values of dementia care, including dignity, respect, empowerment and autonomy (Calkins, 2018; Chaudhury et al., 2013; Hung et al., 2016; Olson and Albensi, 2021). The findings also show that familiarity includes recognizable elements meaningful to the individual or a group.

### 3.1 A homelike environment

A homelike environment was found to be present in 60 % of the publications with definitions associated with a familiar environment. It is important to note that clarity is required in the terms used to describe a homelike environment.

#### 3.1.1 Homelike or domestic

The term “domestic” was replaced or used interchangeably in five papers to describe a homelike atmosphere (Chen et al., 2021; Fisher et al., 2018; Hung et al., 2016; Seetharaman et al., 2022; Verbeek et al., 2012). The data suggests that familiar environments adopting a homelike design may contribute to less stress and confusion, which results in lower levels of agitation (Calkins, 2018; Fisher et al., 2018; Seetharaman et al., 2022). A familiar, homelike environment is also found to be aligned with supporting a person-centered care model and promoting personhood (Calkins, 2018; Chaudhury et al., 2013; Harkin et al., 2020). The inclusive of personal artifacts or belongings was also associated with familiarity, and these elements can be beneficial in aiding navigation, increasing levels of comfort, and enhancing positive engagement with activities of daily living (ADLs) (Bae and Asojo, 2022; Canham et al., 2017; Chen et al., 2021; Fisher et al., 2018; Lee et al., 2021; Olson and Albensi, 2021; Van Hoof et al., 2016).

#### 3.1.2 Small-scale

Another aspect of the design is the need for small-scale residential dwellings (*n* = 16) such as a cottage, small home, or unit (Chaudhury et al., 2013; De Boer et al., 2018). These environments visually encapsulate a non-institutionalized setting and are highly correlative with a homelike environment design associated with the population within the studies (Calkins, 2018; Chen et al., 2021; Danes, 2012; Ellingsen-Dalskau et al., 2021; Fisher et al., 2018; Harkin et al., 2020; Lee et al., 2021; Verbeek et al., 2012).

#### 3.1.3 Technology

Technology was found to be an essential component of creating a meaningful home-like space that enables connections to the world beyond. Residents have requested their own television, as they have in their own homes, or the availability of Wi-Fi technology to enable video communication or access to the internet (Fisher et al., 2018). These technological devices have been found to help residents continue to engage in familiar activities and connect meaningfully to the world outside by being informed of social or worldly events (Chen et al., 2021; Fisher et al., 2018; Olson and Albensi, 2021).

### 3.2 A connection to wellbeing

The evidence supported a strong association with the theme of designing with the wellbeing of the population in mind (Campo and Chaudhury, 2012; Canham et al., 2017; Chaudhury et al., 2018; Danes, 2012; Fisher et al., 2018; Fleming and Purandare, 2010; Garcia et al., 2012; Harkin et al., 2020; Lee et al., 2016; Schmidt et al., 2018; Van Hoof et al., 2016). Factors such as positive psychological, physical, and social connections or relationships are key contributors to the wellbeing framework (Kinderman et al., 2011). A greater sense of social connection or belonging with the world around them is intrinsically linked with living well (Harkin et al., 2020). The three sub-themes comprise findings related to emotional regulating, which is intrinsically linked to mood or behavioral responses, physically engaging in familiar activities, and psychosocial benefits attained from receiving person-centered care because of a familiar environment. One key code within the discussion of wellbeing was “technology”. The need for the environmental design to support technology was woven through all three sub-themes and discussed in a supportive and positive light to improve wellbeing.

#### 3.2.1 Emotional regulation

The design of appropriate spaces or a variety of familiar spaces can aid emotional regulation; reducing responsive behaviors, negative moods, and emotions (Calkins, 2009; Hong and Song, 2009; Hung et al., 2016; Lee et al., 2016). The use of technology to create familiar lighting, be it the level of lighting, design of the lighting, or room temperature, can aid emotional regulation (Chen et al., 2021; Olson and Albensi, 2021). For instance, softer ample lighting that is aligned with the household lighting of the population can help to reduce shadows, and help people to feel calm and safe. Environments that are deemed unfamiliar contribute to safety concerns and impact their wellbeing (Lee et al., 2016; Schmidt et al., 2018; Talebzadeh et al., 2023; Van Hoof et al., 2016). Environments that include long hallways, monotonous design in large facilities, poor acoustics, is unrecognizable features can contribute to spatial disorientation and distress (Lee et al., 2016, 2021; Talebzadeh et al., 2023).

#### 3.2.2 Familiar activities

Design can also contribute to the ability to actively engage in familiar activities, including those associated with maintaining or enhancing interpersonal relationships (Bae and Asojo, 2022; Garcia et al., 2012; Han et al., 2016; Hung et al., 2016; Schmidt et al., 2018). Such interactions positively impact one's social networks, quality of relationships, level of comfort, adaptive coping mechanism, and emotional resilience when navigating daily complexities (Bae and Asojo, 2022; Calkins, 2018; Canham et al., 2017; Chaudhury et al., 2013). Similar to the discussion in a home-like environment and technology, the findings also indicate that due to changing population trends, older adults are now bringing in personal technological devices that are familiar to them for lifestyle purposes or social connections, such as smartphones, tablets, and other wearables (Woodbridge et al., 2018).

#### 3.2.3 Person-centered care

When familiarity is considered in the design within the context of wellbeing, staffs are well-supported to deliver person-centered care (Harkin et al., 2020). With the unique and personalized preferences and history of the person incorporated into the environment, staff working with residents can utilize the space around them to form meaningful emotional, cognitive, and social connections, which can increase environmental mastery for the person with dementia (Calkins, 2018; Canham et al., 2017; Chen et al., 2021; Danes, 2012; Fisher et al., 2018; Lee et al., 2016, 2021; Olson and Albensi, 2021; Tierney et al., 2022). A contemporary example would be an environment that can cater to personalized technology that is tailored to cultural contexts or individual needs and seems to enhance various forms of connection—social, physical, cognitive, and emotional. Personalized technology appears to bridge the relationship between past and present, allowing staff to create an atmosphere that contributes to a sense of identity or emotional security. A familiar design in the form of artifacts, fixtures, and fittings can also support staff to be inclusive of an individual's cultural identity (Douglas and Lawrence, 2015; Fleming and Purandare, 2010; Han et al., 2016). People may use technology to introduce culturally specific or individualized soundscapes for reminiscence or to elicit positive emotions (Douglas and Lawrence, 2015; Talebzadeh et al., 2023).

### 3.3 Multisensory integration within the built environment

The human senses work together to enhance our perception of the world, helping us to understand space and place through communication between neurons in the midbrain and cerebral cortex (Stein and Stanford, 2008). A familiar environment is created when the design supports multisensory integration including sight, temperature, taste, smell, touch, and sound. Dementia care often emphasizes managing symptoms, but creating a familiar sensory environment offers a proactive and preventive approach. A design strategy that focuses on familiarity and multisensory integration can support and enable people with a diverse range and varying levels of cognitive abilities who require multisensory support to flourish (Moyle et al., 2011; Seetharaman et al., 2022).

The design of these spaces within the literature, such as circulation areas, dining rooms, and bathrooms, have been depicted as unfamiliar spaces for mealtime, with literature suggesting poor multisensory integration (Douglas and Lawrence, 2015; Lee et al., 2016). They are often described as spaces that do not visually align with the schema for residents, with unfamiliar levels of sound, textures, and smells and temperature. The combination and amalgamation of exposure to a suite of unfamiliar sensory stimuli at varying levels can contribute to emotional dysregulation (Bae and Asojo, 2022; Wiener and Pazzaglia, 2021). For mealtimes, this results in lower levels of nutritional intake and, in some instances, agitation among residents (Douglas and Lawrence, 2015). Multisensory integration and familiarity significantly impacted ADLs, such as personal health and hygiene, a crucial daily care component. Spaces such as the bathroom and showers were widely discussed in the literature (*n* = 14) with much of the design in these environments being unfamiliar and institutional, and contributing to negative responses such as fear, agitation, or aggression (Bae and Asojo, 2022; Day et al., 2000; Lee et al., 2021; Namazi and Johnson, 1996). Appropriate design elements in these spaces have been noted to promote independence and comfort, and reduce aggression (Bae and Asojo, 2022; Danes, 2012; Lee et al., 2021). A combination of the appropriate application of stimuli to create familiar spaces has been found to be beneficial in conveying sensory information that enables and empowers residents when they are able to recognize the function of the environment (Bae and Asojo, 2022; Calkins, 2018; Motzek et al., 2017; Tao et al., 2018). Without clear integration, the environment appears overwhelming and intimidating, resulting in disabling space when cues are poorly understood.

Multisensory integration is key when supporting the visuospatial function of a person with dementia (Day et al., 2000; Hong and Song, 2009; Tao et al., 2018). Visuospatial function is the ability to identify and define structures, objects, and locations within a space (Fukui and Lee, 2009; Salimi et al., 2018) and comprises of visual perception, memory, and visuoconstruction, which is the ability to make sense of different structures, spaces and shapes. From a neurological perspective, the recognition of a familiar environment is a complex task. Spatial capacity, which is the ability to understand and move within a space, is associated with a familiar environment and within it, feelings of safety (Schmidt et al., 2018). Circulation areas that were too complex resulted in dissatisfaction with the built environment (Tao et al., 2018). It was found that adopting multiple visual cues, such as the use of signage at appropriate heights, photographs, personal artifacts, clocks, wall colors, and visual access to living spaces, can be supportive of individuals who may be living with visuospatial dysfunction (Calkins, 2018; Day et al., 2000; Fisher et al., 2018; Hong and Song, 2009; Tao et al., 2018; Woodbridge et al., 2018).

The findings also included the combination of suitable and appropriate lighting to reduce glare caused by sunlight (Calkins, 2018). Visual cues introduced with multiple familiar visual elements, ample lighting, highly legible text, is utilized by people with dementia (Chen et al., 2021; Hong and Song, 2009; Tao et al., 2018). The literature touches on the need for the location of the bathroom to be clearly visible while maintaining a home-like interior (Calkins, 2018; Chen et al., 2021). Entrances and capacity of rooms for personal hygiene, such as bathrooms and shower areas, are recommended to have a larger spatial capacity (Lee et al., 2016, 2021; Namazi and Johnson, 1996; Olson and Albensi, 2021). A larger space enables the storage of personal belongings or artifacts that promote a sense of familiarity, leading to a sense of recollection, supporting staff who are assisting in personal hygiene tasks. The familiar environment promotes a sense of safety for staff and allows for the safe maneuvering of mobility equipment such as wheelchairs and hoists when residents are calm (Lee et al., 2021; Namazi and Johnson, 1996). Fear is attributed to the sight of unfamiliar hygiene equipment, such as tub lifts and whirlpools (Day et al., 2000; Namazi and Johnson, 1996). However, some solutions were shared within the literature such as pairing familiar cues with the environment, such as a duck with a bath and the implementation of an appropriate tablecloth and flowers to denote lunchtime resulted in an increase in the recollection of these activities and reduced stressors (Woodbridge et al., 2018). The presence of a familiar scale, such as an appropriate size dining table, has been found to contribute to a reduction of aggression, agitation, and anxiety and increased social interaction and quality of life (Chaudhury et al., 2018; De Boer et al., 2018; Olson and Albensi, 2021). Visual clutter, such as unfamiliar medical and food service equipment that do not align with the visual cues of the dining space, will result in a lack of recognition (Douglas and Lawrence, 2015). Some examples found in the literature include appropriate lighting and the use of visual contrast to support spatial capacity and build familiarity with mealtime routines and dining (Douglas and Lawrence, 2015). In dining areas, visual access to familiar items can enhance social engagement among residents and staff by providing a shared opportunity for conversation (Adlbrecht et al., 2021).

Findings have indicated that our brains are intricate in the daily navigation of soundscapes, and dementia has been found to impact the structural aspects of the brain associated with auditory function (Johnson et al., 2021). A noisy environment, which can be unfamiliar and overwhelming for an individual, can result in social disconnection, agitation, stress, and decreased meal intake (Calkins, 2018; Chaudhury et al., 2013; Douglas and Lawrence, 2015; Lee et al., 2016; Moyle et al., 2011). The impact of familiar sounds such as the introduction of familiar music replacing relaxing music, was found to contribute to an increment of 20% in food intake per meal (Douglas and Lawrence, 2015). Familiar soundscapes are also attributed to feelings of safety (Talebzadeh et al., 2023).

Thermal conditions associated with the temperature of the space can influence levels of comfort and perception of the environment. Appropriate temperature control, humidity, and ventilation systems can help create a calming and familiar environment (Day et al., 2000; Lee et al., 2016). A cold unfamiliar bathroom can make an individual feel uncomfortable and vulnerable, which results in discouraging or disabling an individual from carrying out personal hygiene activities (Day et al., 2000). Confined spaces with a high-temperature setting, coupled with poor air ventilation, have also been known to contribute to levels of agitation (Lee et al., 2016). Much of the evidence is attributed to overwhelming stimuli; however, an unfamiliar environment that is sensorily sterile and void of personal and cultural connections can contribute to feelings of restlessness and helplessness (Lee et al., 2021).

## 4 Discussion

Familiarity is a crucial characteristic in designing facilities for residents with dementia. This is reflected in the principle associated with familiarity put forward in the ADI (Fleming et al., 2020) report on dementia, design, and built environments. A familiar environment is associated with positive wellbeing, enablement, increased social engagement, feelings of safety, and reduced responsive behaviors (Schmidt et al., 2018; Talebzadeh et al., 2023). The results show that various design definitions, characteristics, and recommendations associated with familiarity have been published in the last decades. Having only 46 publications over 23 years (Figure 4), presents multiple challenges. The scarcity of studies significantly constrains the opportunity to cultivate a comprehensive and sophisticated understanding of the topic. A sparse publication record results in a lack of perspectives, rigorous critique, all of which impede the advancement of the understanding of familiar environments. The limited volume of studies over an extended timeframe restricts opportunities to contribute to a clear definition of familiarity, resulting in fragmented perspectives which makes it challenging for researchers and stakeholders to engage with the topic meaningfully. Consistent and growing research dissemination is essential for fostering awareness within academic circles and among the public. A limited publication record translates to reduced discourse in academic forums, fewer citations, and diminished visibility, further obstructing the understanding of familiarity. To reiterate, restaurants, family, or buffet-style dining areas can be considered familiar dining spaces as they resemble a space where the population or residents may partake in meals. However, it may not be a familiar space for a daily mealtime activity (Douglas and Lawrence, 2015). As uncovered in the literature, some large dining spaces may contribute to negative stimuli and wellbeing, with environments lacking contextually familiar and population or culturally appropriate designs (Chaudhury et al., 2013; Talebzadeh et al., 2023; Woodbridge et al., 2018). This begs the question of what constitutes or defines familiarity in dementia design, which is the aim of this review.

The findings present themes that emphasize an approach that prioritizes the home, emotional wellbeing, and sensory engagement, contributing to an enhanced quality of life for people with dementia. An important aspect of the findings is the interconnected relationship that is found between themes and it needs to be highlighted because this poses a challenging especially in the implementation of familiarity in design (Marquardt and Schmieg, 2009; Olson and Albensi, 2021; Wiener and Pazzaglia, 2021). When terms have interconnected relationships, it can be challenging to distinguish between specific applications or design elements which may result in ineffective implementation. Examples provided in the findings show that a homelike environment centers on familiarity and contributes to connections to one's wellbeing (Fisher et al., 2018). An environment that resembles home can alleviate feelings of institutionalization, fostering a sense of peace and security, and this has overlapping attributes with the theme of multisensory integration (Calkins, 2018). Being surrounded by familiar elements is crucial to providing solace and reassurance during vulnerable times. Connections to positive wellbeing play a vital role and they are tightly intertwined with one's surroundings. For residents, a well-integrated sensory environment that is home-like and designed with wellbeing in mind can spark connection and joy (Chaudhury et al., 2018; Ellingsen-Dalskau et al., 2021; Fisher et al., 2018; Harkin et al., 2020; Van Hoof et al., 2016). These contextual examples can help to provide a clearer distinction and mitigate the confusion to ensure an effective adoption of the principle of design.

### 4.1 A homelike environment

A salutogenic or a non-institutionalized environment that is homelike is a key theme in the findings as a design characteristic. However, designers must incorporate a homelike design that is relevant and culturally sensitive to the population that will be utilizing the space. The term “homelike” is an important all-encompassing terminology to describe a space associated with comfort, warmth and a connection to one's family home. The term “domestic” was common throughout the literature; however, it may not be an adequate substitute for “homelike.” The term domestic is associated with a space where one carries out activities of daily living (Percival, 2002). The space can be domestic but may not have a salutogenic homelike design. The principle of familiarity considers the individual with dementia or population's context of a homelike environment, and this is inclusive of factors impacting wellbeing, and population-specific multisensory integration of design elements. This definition does not eclipse or overwrite the current principle; however, it supports or assists it rigorously, backed by evidence to enable rational decision-making when ensuring that spaces designed are familiar to specific to the population of people with dementia.

### 4.2 A connection to wellbeing

The wellbeing of the individual and the population is a crucial factor in the design of familiar environments. Reviewing the findings, a familiar environment that embraces the wellbeing of a population considers not just the engagement of the physical needs of the population or the individual but also considers design characteristics that may impact the social, cognitive, emotional and cultural needs. The findings touch on familiar environments that promote enablement and continuing engagement of familiar activities, which leads to a preservation of social citizenship, personhood, and, ultimately, a sense of positive wellbeing. When designing a familiar environment, it would be ideal for designers to consider spaces that allow fair access and inclusion of individuals in familiar daily activities and opportunities allow people to exercise autonomy and agency. The spaces should provide the ability to participate and contribute meaningfully as an individual or as a group. The space should allow individuals to maintain a sense of belonging and empowerment, which will contribute to an individual's wellbeing. Again, it is important to note that there are shared characteristics between the themes of wellbeing and multisensory integration. In both themes, familiar design contributes to population-appropriate stimuli and was found to positively impact cognitive and emotional wellbeing, reducing responsive behaviors.

### 4.3 Multisensory integration within the built environment

In the areas of multisensory integration, the discussion on stimuli is robust and much more defined compared to discussions on home-like environments and wellbeing. Multisensory integration was found to play a significant role in the characteristics of the design, and its impact on stimuli and the behaviors of individuals. This theme suggests that the development of familiar environments requires the designer to examine if the work impacts the different senses and if the design contributes to multi-layered context-dependent cueing that can provide a rich sensory experience (Wiener and Pazzaglia, 2021). Multiple cueing is not a new concept (Day et al., 2000), but when considered in the context of a familiar environment, the need for population or culturally sensitive design is encouraged as there are differences in how populations experience familiar sensory stimuli. This is especially so in design factors that encompass or influence auditory function or thermoregulation (Sun and Fleming, 2018). Population-specific or culturally sensitive design appears to underpin the key themes discussed. The findings indicate a need for co-design work with people or residents with dementia. Adopting co-design when designing familiar environments can strengthen the application of the principle to address cultural sensitivities (Sun, 2020; Wiener and Pazzaglia, 2021). Familiar environments that are co-designed with people with dementia and key stakeholders can help to ensure that the environment is user-centric, fit for purpose, innovative, and appealing.

An additional gap identified was the global distribution of publications on familiarity and dementia design. More studies on populations from Asia and the Middle East are required as most studies stem from Europe, the United States of America, Canada, or Australia. Studies from these regions can help fill the gap in understanding differences in the built environmental design for diverse populations with dementia. From a geographical perspective, these countries have very diverse cultures, utilization of technology, spiritual practices, and climates, which influence the design and culturally homelike environments. Studies from these countries may assist a range of stakeholders with solutions on tackling the implementation of a familiar environment for people or migrants with dementia in an increasingly diverse world.

## 5 Conclusion

Familiarity remains a key principle consistently prevalent in designing environments for people with dementia. While there is a diverse range of characteristics and definitions associated with familiarity, there is a robust association between familiarity and built environmental designs that are homelike. Design considerations such as multisensory integration and the inclusion of the population's wellbeing within the context of a familiar design can enable a successful and sustainable implementation of dementia design. Therefore, designing for familiarity must prioritize a homelike essence tailored to the population, foster a deep connection to their wellbeing, and embrace the richness of multisensory integration to create truly meaningful and impactful spaces.
